# Identification and validation of methylation-driven genes prognostic signature for recurrence of laryngeal squamous cell carcinoma by integrated bioinformatics analysis

**DOI:** 10.1186/s12935-020-01567-3

**Published:** 2020-09-29

**Authors:** Jie Cui, Liping Wang, Waisheng Zhong, Zhen Chen, Jie Chen, Hong Yang, Genglong Liu

**Affiliations:** 1 Department of Pathology, Affiliated Cancer Hospital & Institute of Guangzhou Medical University, Guangzhou, 510095 Guangdong P. R. China; 2 Department of Otorhinolaryngology Head and Neck Surgery, The First Affiliated Hospital of Hainan Medical University, Haikou, 570102 Hainan P. R. China; 3 Department of Head and Neck Surgery, Affiliated Cancer Hospital & Institute of Guangzhou Medical University, Guangzhou, 510095 Guangdong P. R. China; 4 Department of Head Neck Surgery, Hunan Cancer Hospital and The Affiliated Cancer Hospital of Xiangya School of Medicine, Central South University, Changsha, 410000 Hunan P. R. China; 5 Department of Intensive Care Unit, Shunde Hospital, Southern Medical University (The First People’s Hospital of Shunde), Foshan, 528308, Guangdong P. R. China

**Keywords:** Laryngeal squamous cell carcinoma, DNA methylation-driven genes, Epigenetics, Prediction, Recurrence-free survival

## Abstract

**Background:**

Recurrence remains a major obstacle to long-term survival of laryngeal squamous cell carcinoma (LSCC). We conducted a genome-wide integrated analysis of methylation and the transcriptome to establish methylation-driven genes prognostic signature (MDGPS) to precisely predict recurrence probability and optimize therapeutic strategies for LSCC.

**Methods:**

LSCC DNA methylation datasets and RNA sequencing (RNA-seq) dataset were acquired from the Cancer Genome Atlas (TCGA). MethylMix was applied to detect DNA methylation-driven genes (MDGs). By univariate and multivariate Cox regression analyses, five genes of DNA MDGs was developed a recurrence-free survival (RFS)-related MDGPS. The predictive accuracy and clinical value of the MDGPS were evaluated by receiver operating characteristic (ROC) and decision curve analysis (DCA), and compared with TNM stage system. Additionally, prognostic value of MDGPS was validated by external Gene Expression Omnibus (GEO) database. According to 5 MDGs, the candidate small molecules for LSCC were screen out by the CMap database. To strengthen the bioinformatics analysis results, 30 pairs of clinical samples were evaluated by digoxigenin-labeled chromogenic in situ hybridization (CISH).

**Results:**

A total of 88 DNA MDGs were identified, and five RFS-related MDGs (LINC01354, CCDC8, PHYHD1, MAGEB2 and ZNF732) were chosen to construct a MDGPS. The MDGPS can effectively divide patients into high-risk and low-risk group, with the area under curve (AUC) of 0.738 (5-year RFS) and AUC of 0.74 (3-year RFS). Stratification analysis affirmed that the MDGPS was still a significant statistical prognostic model in subsets of patients with different clinical variables. Multivariate Cox regression analysis indicated the efficacy of MDGPS appears independent of other clinicopathological characteristics. In terms of predictive capacity and clinical usefulness, the MDGPS was superior to traditional TNM stage. Additionally, the MDGPS was confirmed in external LSCC cohorts from GEO. CMap matched the 9 most significant small molecules as promising therapeutic drugs to reverse the LSCC gene expression. Finally, CISH analysis in 30 LSCC tissues and paired adjacent normal tissues revealed that MAGEB2 has significantly higher expression of LSCC compared to adjacent non-neoplastic tissues; LINC01354, CCDC8, PHYHD1, and ZNF732 have significantly lower expression of LSCC compared to adjacent non-neoplastic tissues, which were in line with bioinformatics analysis results.

**Conclusion:**

A MDGPS, with five DNA MDGs, was identified and validated in LSCC patients by combining transcriptome and methylation datasets analysis. Compared TNM stage alone, it generates more accurate estimations of the recurrence prediction and maybe offer novel research directions and prospects for individualized treatment of patients with LSCC.

## Background

As an aggressively malignant neoplasm, laryngeal squamous cell carcinoma (LSCC) is one of the most prevalence cancers in head and neck region, and represents 85–95% of all laryngeal cancer [1]. According to the American Cancer Society, the estimated respective new cases and new death are 13 150 and 3 710 annually, with incidence and mortality rates of 4.0 and 1.1 per 100 000, respectively [2]. Current multimode treatments, including surgery, radiotherapy, chemotherapy, targeted therapy and so on, are applied to cure LSCC patients [3]. Though treatments have improved during the past decades, long-term survival is hampered as more than 40% of LSCC patients experience disease recurrence approximately at 5 years after radical treatment [4].

Hence, identifying accurate predictive models and reliable biomarkers to screen out which subset of patients with LSCC is apt to develop recurrence is urgently needed, which help to optimize therapeutic strategies and exploit valuable molecular targeted therapy in LSCC patients.

LSCC is a heterogeneous disease in terms of therapeutic response and clinical prognosis. To a certain extent, clinical heterogeneity can be related to distinct molecular subtypes by gene expression pattern [5].RNA expression profiles usually exhibits relative stochastic and rapid variations, which can be directly related to important pathways in malignant cells. DNA methylation, serves as a major epigenetic modification that is involved in the transcriptional regulation of genes and maintains the stability of the genome, is less variable and semi-stable, but show large variations linked to the activity of cellular processes. Therefore, the combination of transcriptome and epigenetic status would be helpful to identify new markers and improve the accuracy of recurrence prediction. What’s more, changes in DNA methylation with a high level of plasticity allows tumor cells to quickly adapt to changes in metabolic restrictions or physiology during the process of tumorigenesis [6, 7]. Hence, it is reasonable to analyses the DNA methylation pattern in the tumor cells in order to find predictors for the recurrence and novel therapeutic targets in LSCC patients.

The availability of high throughput genomic assays such as RNA-seq and DNA methylation-seq have opened the possibility for a comprehensive characterization of all molecular alterations of cancers, leading to the discovery of new biomarkers of clinical and therapeutic value [8]. In present study, we performed a genome-wide integrated analysis of methylation and the transcriptome to characterize the crosstalk between DNA methylation and RNA regulation for patients with LSCC in The Cancer Genome Atlas (TCGA) database. We identify methylation-driven genes (MDGs), and then developed a methylation-driven genes prognostic signature (MDGPS) capable of predicting the recurrence-free survival (RFS), and further screen correlated small molecule target drugs. The proposed MDGPS was validated in external datasets from the GEO database. In additional, we assessed the predictive ability and clinical application of the MDGPS and compared it to the TNM stage.

## Materials and methods

### Sample selection and data processing

We downloaded DNA methylation (111 LSCC samples and 12 normal samples), RNA-sequencing profiles (117 LSCC samples and 16 normal samples) and clinical information data (Additional file 1: Material S1) of LSCC patients from TCGA (https://gdc.cancer.gov/), which recorded before February 14, 2020. Excluding the unavailability of DNA methylation or gene transcriptome datasets or RFS time < 1 month, as a result, a total of 81 LSCC patients were enrolled in this analysis. The GSE27020 microarray dataset (https://www.ncbi.nlm.nih.gov/geo/query/acc.cgi?acc=GSE27020) comprises 109 LSCC specimens with gene expression profiles and the associated clinical characteristics **(**Additional file 2: Material S2**).**GSE25727 microarray dataset (https://www.ncbi.nlm.nih.gov/geo/query/acc.cgi?acc=GSE25727) includes 56 LSCC specimens with gene expression profiles and the corresponding prognostic information (Additional file 3: Material S3). The clinical end point was RFS, defined as time from final surgical excision to recurrence. Patients not having a recurrence or those patients who died without recurrence at last follow-up are considered as censored observations. All data were normalized in the R computing environment using the edgeR package or Limma package. Methylation data were in form of β value, representing the ratio of the methylation probe data vs total probe intensities. Then, the average DNA methylation value for all CpG sites correlated with a gene was calculated and merged into a matrix with the function of TCGA-Assembler. Data were utilized according to the data access policy of TCGA and GEO. All analyses were conducted in accordance with relevant regulations and guidelines.

Another in-house dataset including 30 LSCC tissues and paired adjacent normal tissues were collected from patients who underwent surgery at Affiliated Cancer Hospital & Institute of Guangzhou Medical University between January 2016 and December 2019. No recruited patients received any preoperative treatment. The adjacent non-cancerous tissues were collected > 2 cm from the tumor margins on the same or another lobe. All tissue samples were blocked of formalin-fixed paraffin-embedded material and stored at 2–8 °C with desiccation until use for later experiments. The studies involving human tissues samples were reviewed and approved by the Research Ethics Committee of Affiliated Cancer Hospital & Institute of Guangzhou Medical University, and complied with the Declaration of Helsinki. All patients were aware of the present study and signed an informed consent agreement.

### Identification of DNA methylation-driven genes

The MethylMix R package was employed for analysis that integrated DNA methylation data for 117 LSCC samples and 16 normal samples and paired gene expression data for 111 LSCC samples to appraise DNA methylation events that have a significant impact on the expression of the corresponding gene, indicating that the gene is a DNA methylation-driven gene (MDG). A total of three procedures of MethylMix analysis were described as previous studies [9].In additional, the differential methylation (DM) value where gene with a positive DM value of signifies hypermethylation and gene with a negative DM value signifies hypomethylation can be applied in subsequent analysis.

### Functional enrichment and pathway analysis of methylation-driven genes

Gene ontology (GO) analysis, including the molecular function (MF), biological process (BP) and cellular component (CC), was performed on identified MDGs using the DAVID database (https://david.abcc.ncifcrf.gov/),which provides integrative and systematic annotation tools for uncovering biological meaning of genes. And we used GOplot R package to visualize the result. Additionally, pathway analysis was carried out for the MDGs with ConsensusPathDB (https://cpdb.molgen.mpg.de/), which is a functional molecular interaction database, integrating information on gene regulation, signal transduction, biochemical metabolism, protein interacting, genetic interacting signaling in humans. Humancyc, Kegg, Reactome, Wikipathways, Smpdb, and Biocarta and Signalink was selected for subsequent analysis. *P* < 0.05 was set as the threshold value.

### Construction and verification of an MDGPS

First, univariable Cox regression analysis is utilized to select RFS-related MDGs with *P* < 0.05 as the threshold. After primary filtering, the RFS-related MDGs were all assembled into multivariate Cox regression model, then a MDGPS based on these RFS-related MDGs is developed.

The MDGPS risk score was generated through linear combination of the expression levels of independent DNA MDGs using coefficients from multivariate Cox regression as the weights. On the basis of the median risk score, LSCC patients were classified into two cohorts, high-risk cohorts and low-risk cohorts. Survival differences between high-risk group and low-risk group were assessed by Kaplan–Meier survival analysis and then compared by the log-rank test. Time-dependent receiver operating characteristic (ROC) curves by means of the timeROC package were applied to evaluate predictive performance. In addition, stratified analysis base on various clinical characteristics is conducted to evaluate the discrimination ability of MDGPS. Importantly, the GSE27020 and GSE25727 from the GEO database were applied to validate the predictive value of the MDGPS.

### Gene set enrichment analysis

We downloaded GSEA software from the GSEA website (https://software.broadinstitute.org/gsea/index.jsp). 81 LSCC patients were categorized into high-risk cohorts and low-risk cohorts, which was served as the phenotypes. Gene sets related to biosignaling on MSigDB (https://software.broadinstitute.org/gsea/downloads.jsp) could be found on the GSEA home as reference gene sets. Each analysis was repeated 1000 times according to the default weighted enrichment statistical method. Statistically significant pathways were screened based on the false discovery rate (FDR) < 0.25, |enriched score|> 0.35, and gene size ≥ 35 as the cutoff criteria.

### Independence of the MDGPS from clinicopathological features

We carried out univariate and multivariate Cox regression analyses to adjudicate whether the predictive ability of the MDGPS may be independent of other clinicopathological characteristics (including smoke history, alcohol history, age, sex, number of positive lymph nodes (LNs), number of LNs, lymph node ratio (LNR), margin status, lymphovascular invasion, histologic grade, TNM stage, N status, T stage, mutation count, fraction genome altered) of LSCC patients in TCGA database.

Additionally, we validate whether the MDGPS remain be independent of other clinical features in GSE27020 database (age, sex, including smoke history, alcohol history, histologic grade, TNM stage, Radiation therapy).

### Comparison of predictive performance and clinical usefulness between MDGPS and TNM stage

ROC analysis by means of the survivalROC package was carried out to investigate and compare the discrimination ability of the MDGPS signature with traditional TNM staging in TCGA and GSE27020 database. Decision curve analysis (DCA) by means of the stdca.R was employed to evaluate the clinical usefulness and net benefit of the MDGPS, and compared to TNM stage in TCGA and GSE27020 database [10].

### Joint survival analysis and methylated loci associated with RFS

To further investigate the impact of RFS-related MDGs on LSCC patient, we carried out joint survival analysis combined methylation and gene expression to identify hub genes associated with prognosis in patients with LSCC by the survival R package. In additional, we performed the comparative studies between single factor (methylation or gene expression only) and dual factors (methylation + gene expression) in prediction of RFS. Finally, we retrieved relevant loci for RFS-related MDGs from downloaded LSCC methylation data. We merged the value of corresponding methylated sites into one matrix and conducted univariate Cox analysis to select potential prognosis methylated loci. *P* < 0.05 was regarded as statistically significant.

### Identification of candidate small molecules agents

The CMap database (https://www.broadinstitute.org/cmap/), which collects more than 7,000 gene expression profile changes induced by various small molecular drugs, was adopted to investigate candidate small molecules agents for LSCC treatment. 5 RFS-related MDGs were divided into down-regulated (*vs* normal samples based on gene expression level) and up-regulated (*vs *normal samples based on gene expression level) groups, which were uploaded into CMap database to screen related active small molecules agents. Then, the enrichment scores were calculated, which signify similarity rang from − 1 to 1. A negative connectivity score (closer to − 1) indicated higher similarity between the genes, which represents potential therapeutic value, whereas a positive connectivity score (closer to + 1) demonstrate the matched small molecule can induce the state of LSCC cells. The candidate small molecules agents (*P* < 0.01, N > 4, and Enrichment < 0) for anti-LSCC were selected.

### Chromogenic in situ hybridization (CISH)

To validate LINC01354, CCDC8, PHYHD1, MAGEB2 and ZNF732 expression in LSCC, digoxigenin (DIG)-labeled CISH was performed on 30 pairs of tumor and para-carcinoma tissues. The samples were fixed using 4% paraformaldehyde (DEPC, Servicebio) for 2–12 h. Paraffin sections were prepared to perform the hybridizations. Then, the sections were placed in boiling water for 15 min and cooled at room temperature. The specimens were incubated at 37 °C for 30 min in 20 µg/ml Proteinase K (Servicebio) and then rinsed three times in PBS (Servicebio). Prehybridization was conducted at 37 °C for 1 h in hybridization buffer (Servicebio). Then, the prehybridization buffer was replaced with fresh hybridization buffer containing 8 ng/ml of the corresponding probe, and the specimens were incubated at 37 °C overnight. The washed specimens were incubated at room temperature in blocking serum containing BSA for 30 min and then incubated at 37 °C for 40 min with anti-DIG/AP antibody (Jackson).

Staining score given by the two independent investigators were averaged for further comparative evaluation of LINC01354, CCDC8, PHYHD1, MAGEB2 and ZNF732 expression. Tumor cell proportion was scored as follows: 0 (no positive tumor cells); 1 (< 10% positive tumor cells); 2 (10–35% positive tumor cells); 3 (35–70% positive tumor cells) and 4 (> 70% positive tumor cells). Staining intensity was graded according to the following criteria: 0 (no staining); 1 (weak staining, light yellow); 2 (moderate staining, yellow brown) and 3 (strong staining, brown). Staining score was calculated as the product of staining intensity score and the proportion of positive tumor cells. Based on this method of assessment, LINC01354, CCDC8, PHYHD1, MAGEB2 and ZNF732 expression in LSCC tissues and paired adjacent normal tissues was evaluated by the staining score, with scores of 0, 1, 2, 3, 4, 6, 8, 9 or 12. Score was compared by paired t-test in different two groups.

### Statistical analysis

R software (R version 3.5.2) and SPSS statistics 22.0 were utilized to conduct the statistical analysis. A two sided *P* < 0.05 would be recognized as statistically significant except for where a certain *P* value has been given. Additionally, we set a table, including the objective, method and Package name, for using different R packages for analysis (Additional file 4: Table S1).

## Results

### Identification of MDGs, functional enrichment and pathway analyses

Based on three matrices and steps of MethylMix, in total, 88 genes, 77 hypermethylated genes and 11 hypomethylated genes, were defined as the epigenetic drivers with Cor <  − 0.3, |logFC|> 0and P < 0.05**(**Additional file 5: Material S4**)**. The GO term enrichment analysis for MDGs shows the top 6 clusters of enriched sets with significant differences (*P* < 0.05) **(**Additional file 6: Figure S1).As to molecular function (MF), MDGs were mainly involved in nucleic acid binding, metal ion binding, and transcription factor activity, sequence-specific DNA binding. For biological processes (BP), MDGs were mainly enriched in regulation of transcription, DNA-templated and transcription, DNA-templated. As to cellular component (CC), MDGs were mainly involved in (CC) intracellular. The results of pathway enrichment analysis indicated that MDGs were most involved in gene expression (transcription), RNA polymerase II transcription and generic transcription pathway (Additional file 7: Figure S2).

### Construction and verification of an MDGPS

**Fig. 1:**
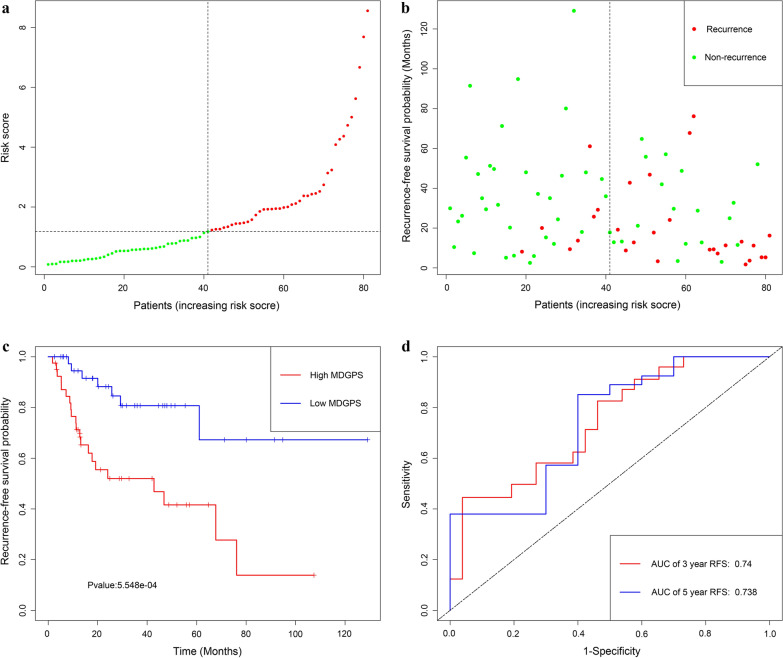
Development of MDGPS for prediction of recurrence in LSCC patients in TCGA database. **a**, **b** Distribution of MDGPS risk score. **c** Time-independent ROC curves with AUC values to evaluate predictive efficacy of MDGPS risk score. **d** Kaplan–Meier estimates of patients' recurrence status and time using the median risk score cut-off which divided patients into low-risk and high-risk groups

**Fig. 2:**
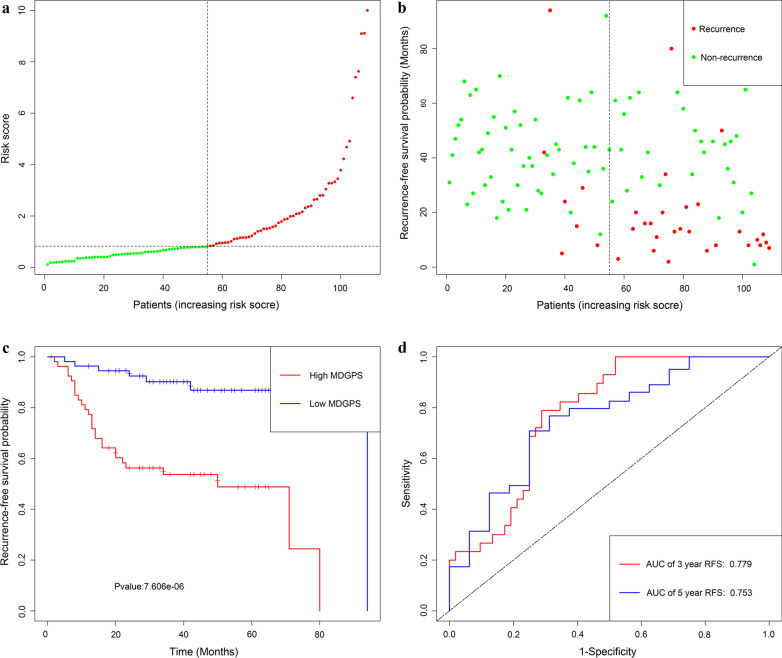
Development of MDGPS for prediction of recurrence in LSCC patients in GSE27020 dataset. **a**, **b** Distribution of MDGPS risk score. **c** Time-independent ROC curves with AUC values to evaluate predictive efficacy of MDGPS risk score. **d** Kaplan–Meier estimates of patients' recurrence status and time using the median risk score cut-off which divided patients into low-risk and high-risk groups

**Fig. 3:**
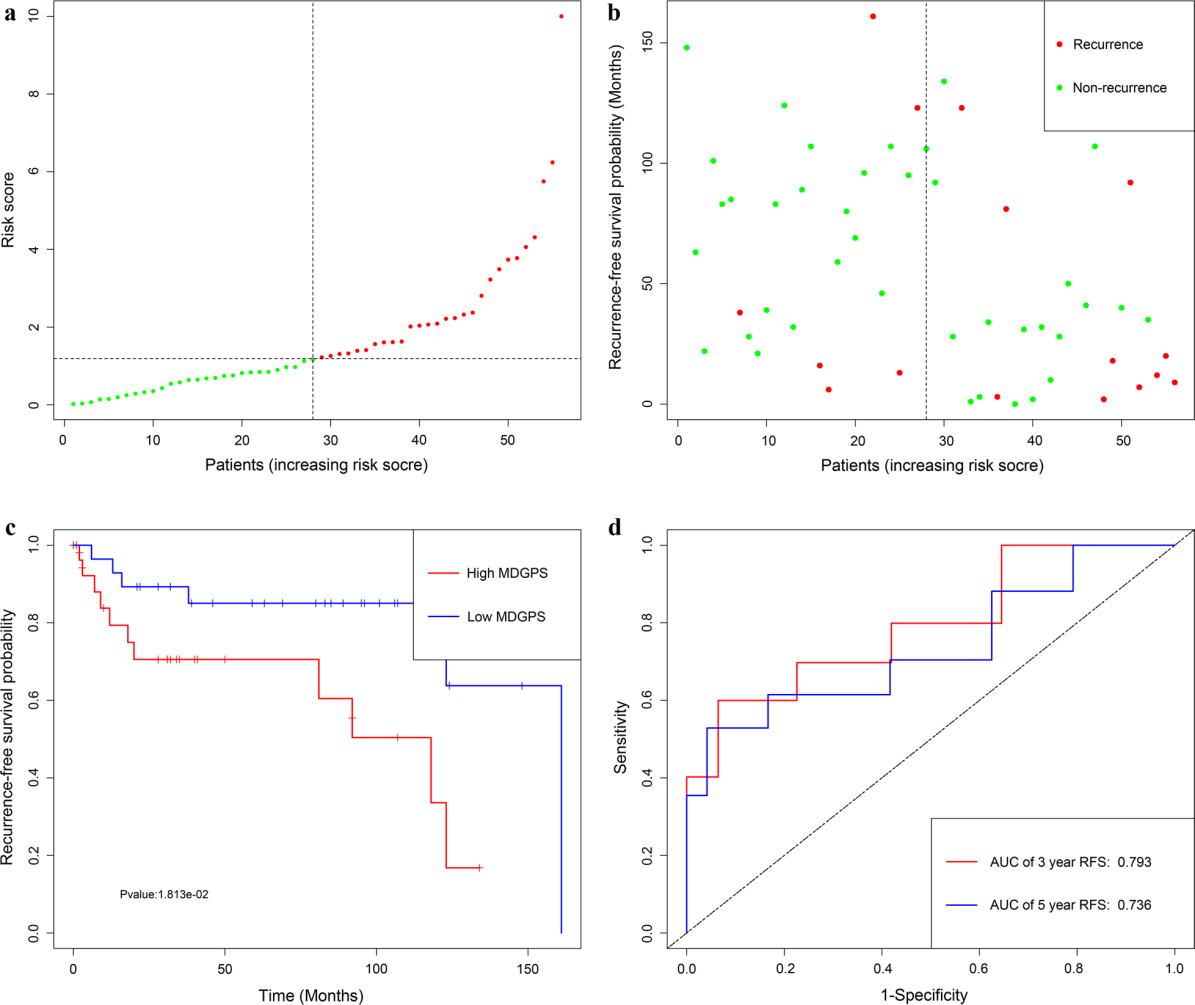
Development of MDGPS for prediction of recurrence in LSCC patients in GSE25727 dataset. **a**, **b** Distribution of MDGPS risk score. **c** Time-independent ROC curves with AUC values to evaluate predictive efficacy of MDGPS risk score. **d** Kaplan–Meier estimates of patients' recurrence status and time using the median risk score cut-off which divided patients into low-risk and high-risk groups

Utilizing the univariate Cox regression analysis, we identify 5 genes, namely LINC01354, ZNF732, CCDC8, PHYHD1 and MAGEB2, associated with RFS with *P* < 0.05 from 88 MDGs **(**Additional file 5: Material S4**)**. The methylation degree distributions of the 5 RFS-related MDGs are displayed in Additional file 8: Figure S3 by methylation β mixed model. Additional file 9: Figure S4 visualized methylation degrees of LINC01354, ZNF732, CCDC8, PHYHD1 and MAGEB2 were negatively correlated with respective expressions in LSCC. An MDGPS was constructed based on 5 RFS-related MDGs, which were all embedded into multivariate Cox regression model. The MDGPS risk score was computed as follows: risk score = (− 0.3206 expression level of LINC01354) + ( − 0.2638 expression level of ZNF732) + ( − 0.2291 expression level of CCDC8) + ( − 0.0102 expression level of PHYHD1) + (0.0167 expression level of MAGEB2). A median cut-off risk score was employed to classify LSCC patients into a high-risk cohorts (n = 40) and a low-risk cohorts (n = 41) in TCGA database (Fig. 1a). Intuitively, Fig. 1b the number of recurrence was significantly lower in the low-risk cohorts compared with high-risk cohorts. The Kaplan–Meier analysis indicated that in low risk cohorts LSCC patients were more inclined to higher RFS time than patients in high risk cohorts (*P* < 0.001) (Fig. 1c). Time-dependent receiver operating characteristic (ROC) curves showed that MDGPS had a superior prediction capacity, with AUC of 0.738 (5 year RFS) and AUC of 0.74 (3 year RFS) (Fig. 3d).In additional, stratification analysis were carried out in subsets of patients with different clinical variables (lymphovascular invasion vs no lymphovascular invasion, positive margin status vs negative margin status, G1-G2 vs G3-G4, I-II stage vs III-IV stage, N0 vs N1–N3, T1–T2 vs T3–T4) for MDGPS. In lymphovascular invasion or no lymphovascular invasion, negative margin status, G1–G2 or G3–G4, III-IV stage, N0 or N1–N3, T3–T4 subgroup, the MDGPS was still a statistically and clinically prognostic model (Additional file 10: Figure S5 and Additional file 11: S6). But, in positive margin status, I–II stage and T1–T2 subgroup did not reach significant statistic. Noteworthily, external GEO cohorts (GSE27020 and GSE25727 database) were utilized to verify the predictive performance of the MDGPS. As was displayed in Figs. 2, 3, patients with low risk score were more prone to survival and had higher RFS time than patients with high-risk score, which consistent with the results of the TCGA dataset. Furthermore, the AUC of MDGPS (AUC of 5 year RFS: 0.753 and AUC 3 year RFS: 0.779 in GSE27020 dataset, AUC of 5 year RFS: 0.736 and AUC 3 year RFS: 0.793 in GSE25727 dataset) confirmed that the predictive accuracy of the prognostic model was satisfactory.

### Gene set enrichment analysis

**Fig. 4:**
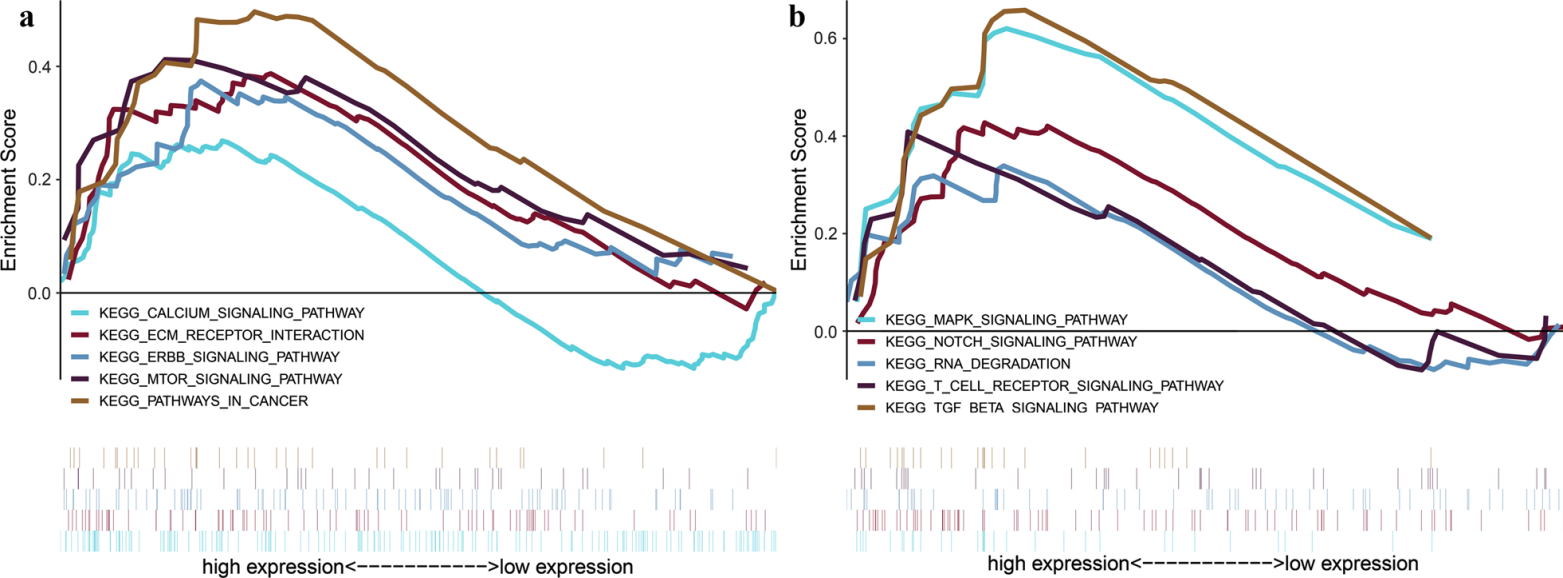
Gene set enrichment analysis for identification of the underlying pathways using risk score as the phenotype (**a**, **b**)

To investigate potential biological pathways for 5 RFS-related MDGs, we carried out the GSEA, and reveal a total of 48 items were significantly enriched with FDR < 0.25. The level of risk score for MDGPS was considered as the phenotypes, and the findings uncovered that high-risk level of MDGPS may closely correlated with several important crosstalk, comprising of calcium signaling pathway, ECM receptor interaction, ErbB signaling pathway, mTOR signaling pathway, pathways in cancer, MAPK signaling pathway, Notch signaling pathway, RNA degradation, TGF beta signaling pathway (Fig. 4).

### Independence of the MDGPS from clinicopathological features

To investigate whether the MDGPS is independent of the clinicopathological characteristics in TCGA database, Univariate Cox regression analysis found that positive margin status, and high MDGPS risk score were associated with shorter RFS 1). Multivariate Cox regression analysis continued to verify that MDGPS was an independent predictor of unfavorable RFS (HR: 1.29, 95% CI 1.06–1.60, *P* = 0.010), after adjustment for other risk variables. In additional, external GEO cohorts (GSE27020 database) were utilized to validate whether the MDGPS is also independent of other clinical features. Univariate and multivariate Cox regression analyses were performed, which indicated that the MDGPS a significant independent indicator for RFS (HR: 1.17, 95% CI 1.09–1.22, *P *< 0.001) by adjusting covariates (Table 2).

**Table 1:** Univariable and multivariable Cox regression analysis for prediction of RFS in TCGA database

Factors	Subgroup	Univariable analysis		Multivariable analysis	
		HR(95% CI)	*P*	HR(95% CI)	*P*
Age		0.96 (0.92–1.00)	0.094	NA	NA
Sex	Female	1			
	Male	0.85 (0.26–2.85)	0.795	NA	NA
Smoking history	No	1			
	Yes	1.47(0.69–3.10)	0.319	NA	NA
Alcohol history	No	1			
	Yes	1.21 (0.51–2.90)	0.668	NA	NA
Number of Lymph nodes		0.99 (0.97–1.00)	0.305	NA	NA
Number of positive LNs		1.00 (0.94–1.05)	0.878	NA	NA
Lymph node ratio		2.02 (0.37–10.95)	0.417	NA	NA
Margin status	Negative	1		1	
	Positive	6.24(2.53–16.28)	0.000*	3.45(1.05–11.35)	0.042*
Lymphovascular	No	1			
invasion	Yes	2.00(0.71–5.66)	0.192	NA	NA
Tumor grade	G1–G2	1			
	G3–G4	0.97(0.43–2.20)	0.942	NA	NA
Clinical T	T1–T2	1			
	T3–T4	0.96(0.29–3.23)	0.949	NA	NA
Clinical N	N0	1			
	N1–N3	1.86(0.84–4.12)	0.123	NA	NA
Clinical stage	I–II	1			
	III–IV	0.53(0.007–4.02)	0.540	NA	NA
Mutation count		0.99(0.98–1.01)	0.185	NA	NA
Fraction Genome altered		0.73(0.07–7.43)	0.788	NA	NA
MDGPS		1.43(1.23–1.67)	0.000*	1.29 (1.06–1.60)	0.010*

*HR* hazard ratio, *CI* confidence intervals, *RFS* recurrence-free survival, *NA* not available

These variables were eliminated in the multivariate cox regression model, so the HR and *P* values were not available. *P < 0.05

**Table 2:** Univariable and multivariable Cox regression analysis for prediction of RFS in GSE27020 dataset

Factors	Subgroup	Univariable analysis		Multivariable analysis	
		HR (95% CI)	*P*	HR (95% CI)	*P*
Age		1.02(0.99–1.06)	0.193	NA	NA
Sex	Female	1			
	Male	21.94(0.34–32.16)	0.377	NA	NA
Smoking history	No	1			
	Yes	20.53(0.06–28.44)	0.679	NA	NA
Alcohol history	No	1			
	Yes	1.17 (0.58–2.33)	0.664	NA	NA
Tumor grade	G1–G2	1			
	G3–G4	0.90(0.64–1.26)	0.998	NA	NA
Clinical stage	I–II	1			
	III-IV	1.00(0.61–1.65)	0.525	NA	NA
Radiation therapy	Yes			NA	NA
	No	2.22(0.98–4.99)	0.055	NA	NA
MDGPS		1.15(1.09–1.22)	0.000*	1.17(1.09–1.24)	0.000*

*HR* hazard ratio, *CI* confidence intervals, *RFS* recurrence-free survival, *NA* not available

These variables were eliminated in the multivariate cox regression model, so the HR and *P* values were not available.*P < 0.05

### Comparison of predictive performance and clinical usefulness between MDGPS and TNM stage

**Fig. 5:**
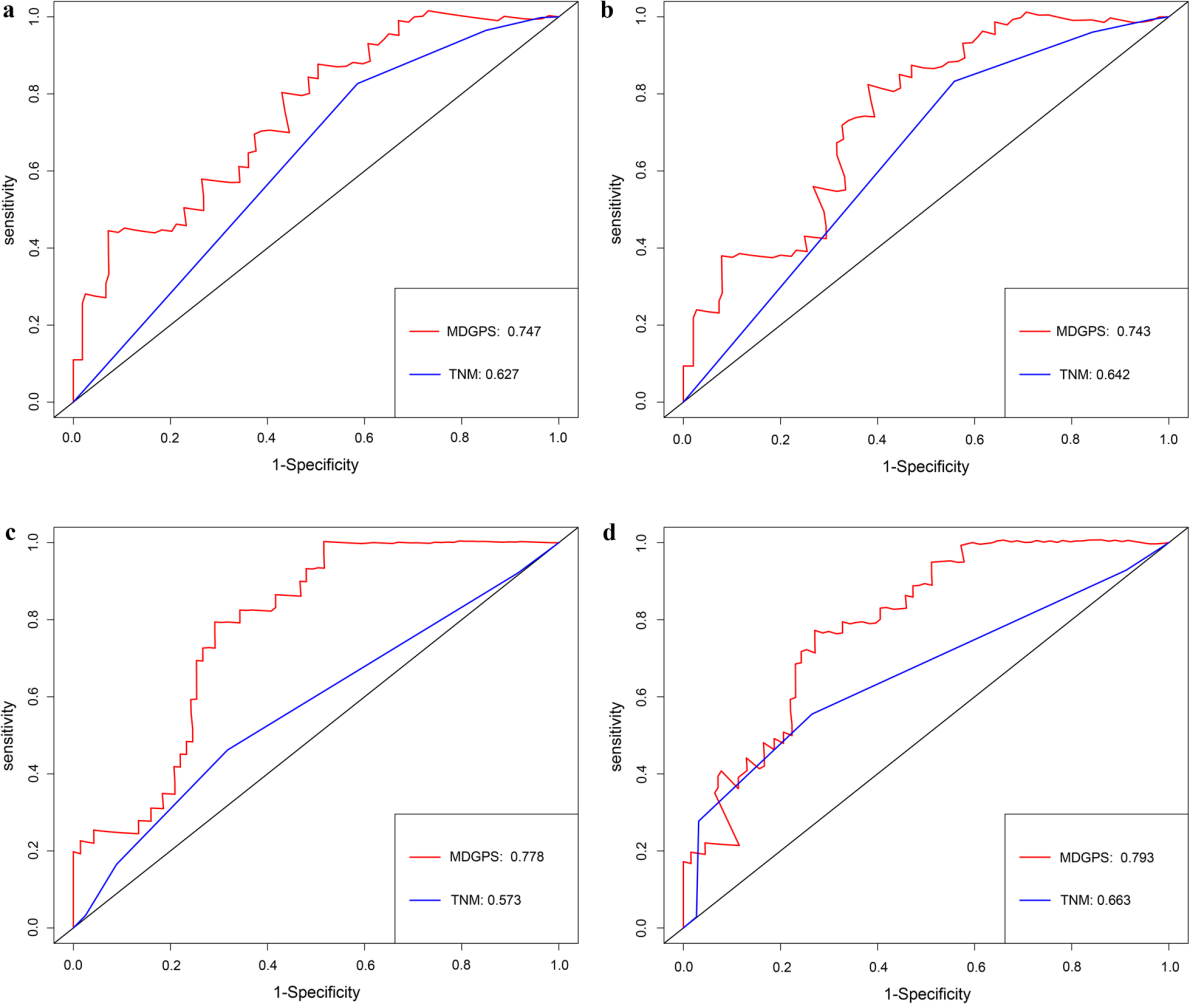
A and B: ROC curves compare the prognostic accuracy of the MDGPS with TNM staging in predicting 3-year (**a**) and 5-year (**b**) recurrence probability in the TCGA dataset. C and D: ROC curves compare the prognostic accuracy of the MDGPS with TNM staging in predicting 3-year (**c**) and 5-year (**d**) recurrence probability in the GSE27020 dataset

**Fig. 6:**
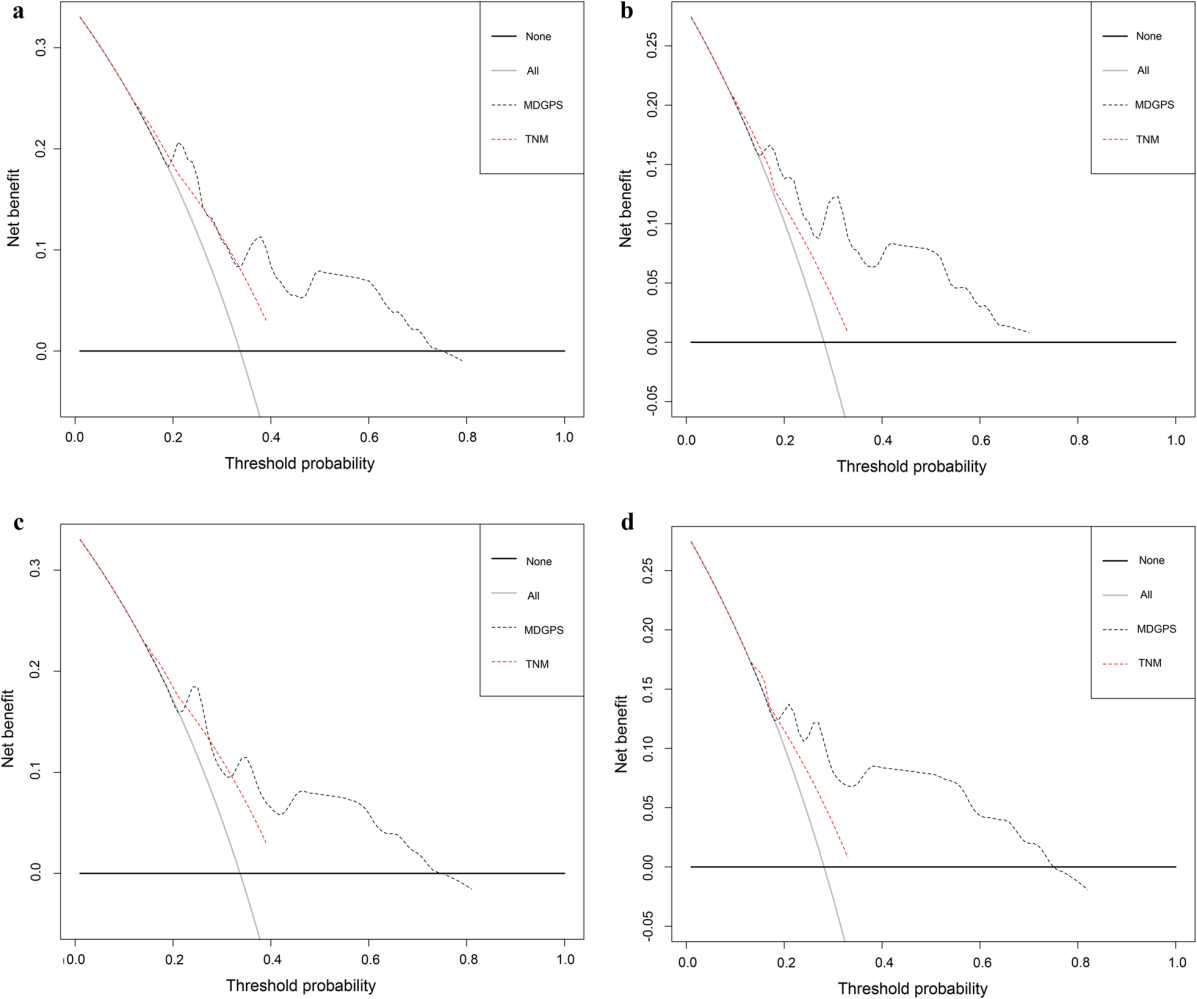
Decision curve analysis for the MDGPS and TNM staging in predicting 3-year (**a**) and 5-year (**b**) recurrence probability in the TCGA dataset. Decision curve analysis for the MDGPS and TNM staging in predicting 3-year (**c**) and 5-year (**d**) recurrence probability in the GSE27020 dataset

To evaluate the predictive ability of MDGPS, we compared MDGPS to AJCC TNM stage model, ROC curve analysis was performed in TCGA database. As was displayed in Fig. 5a, b, the AUC of MDGPS for predicting 5- year and 3-year RFS were 0.743 and 0.747, respectively, while that of TNM stage model were 0.642 and 0.627, respectively. Similar results were also found in the GSE27020 cohorts. The AUC of MDGPS for predicting 5- year and 3-year RFS were 0.793 and 0.778, respectively, and the AUC of the TNM stage model were 0.663 and 0.573, respectively (Fig. 5c, d). Finally, the clinical usefulness of the MDGPS was measured by the DCA, an abstract statistical concept, which provided visualized information on the clinical value of a model. DCA graphically revealed that the MDGPS at diverse cutoff times (5- year and 3-year RFS) was superior to the traditional TNM staging based on the continuity of potential death threshold (x-axis) and the net benefit of risk stratification using the model (y-axis) in TCGA cohorts and GSE27020 cohorts (Fig. 6).

### Joint survival analysis and methylated loci associated with RFS

**Fig. 7:**
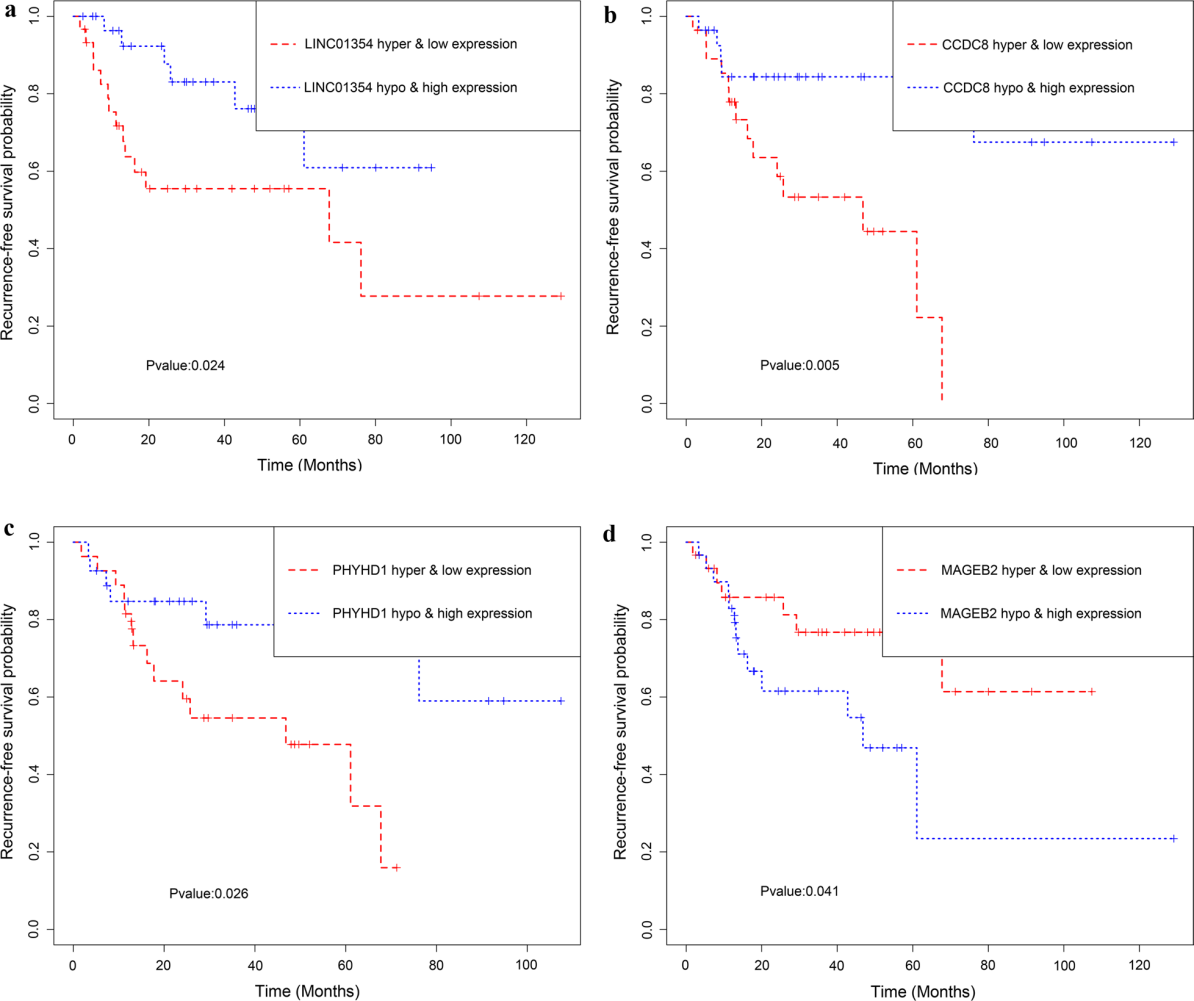
Kaplan–Meier survival curves for the joint survival analysis. **a** The combination of gene LINC01354 methylation and expression. **b** The combination of gene CCDC8 methylation and expression. **c** The combination of gene PHYHD1 methylation and expression. **d** The combination of gene MAGEB2 methylation and expression

**Fig. 8:**
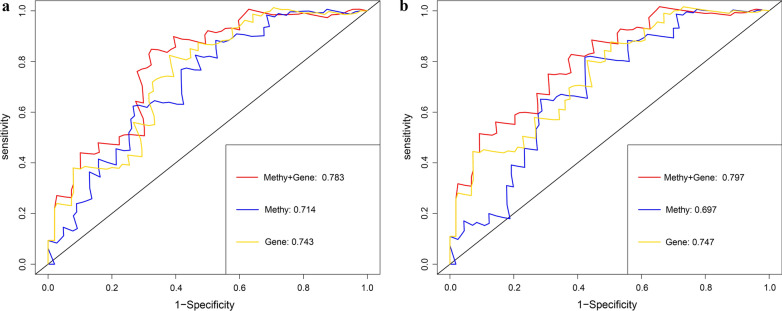
ROC curves compare the prognostic accuracy of the methylation + gene expression with methylation or gene expression only in predicting 3-year (**a**) and 5-year (**b**) recurrence probability

**Fig. 9:**
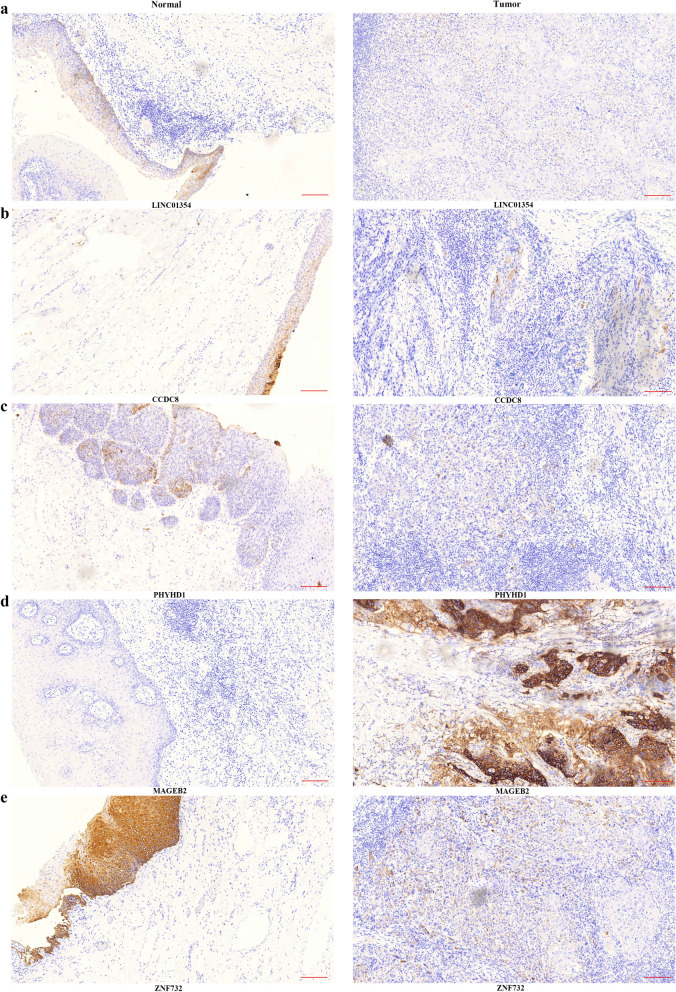
LINC01354 (**a**), CCDC8 (**b**), PHYHD1 (**c**), MAGEB2 (**d**) and ZNF732 (**e**) expression in LSCC tumor specimens and paired adjacent non-tumor tissues was detected by CISH. Scale bars of 100 × magnification, 200 μm

Joint survival analysis, that is, the methylation and gene expression matched evaluation, was additionally conducted to exploit the prognostic value of LINC01354, ZNF732, CCDC8, PHYHD1 and MAGEB2. The hypermethylation and low-expression of LINC01354, CCDC8 and PHYHD1 exhibited a markedly correlation with the poor prognosis of patients with LSCC (Fig. 7a–c); The hypomethylation and high-expression of MAGEB2 exhibited a conspicuous association with the unfavorable prognosis of patients with LSCC (Fig. 9d). Nevertheless, the combination of methylation and expression of ZNF732 did not have a significant effect on prognosis of LSCC patients (Additional file 12: Figure S7). As were shown in Fig. 8a, b, combination of methylation and gene expression data (AUC of 5-year = 0.797 and AUC of 3-year = 0.783) showed a superior prediction RFS ability in comparison to using methylation data only (AUC of 5-year = 0.697 and AUC of 3-year = 0.714) or gene expression data (AUC of 5-year = 0.747 and AUC of 3-year = 0.743). A total of 50 methylation sites (6 methylated sites in LINC01354, 15 methylated sites in ZNF732, 10 methylated sites in CCDC8, 9 methylated sites in PHYHD1, and 10 methylated sites in MAGEB2) were screened out and the univariate Cox regression analysis uncovered that 16 key methylation loci (5 specific methylation sites in LINC01354, 5 specific methylation sites in CCDC8, specific methylation sites in PHYHD1 and 1 specific methylation sites in MAGEB2) were significantly associated with LSCC prognosis (Additional file 13: Table S2).

### Identification of related active small molecules

A total of 3587 small molecules drugs were matched by CMap database **(**Additional file 14: Material S5). Table 3 displayed the 9 significant small molecules agents corresponding to gene expression changes of LSCC after strict selection. Among these small molecules agents, thiocolchicoside (enrichment score =  − 0.85), gibberellic acid (enrichment score =  − 0.714) and ciclacillin (enrichment score =  − 0.866) were related to a highly significant negative fraction and possess potential to reverse the tumor status of LSCC.

**Table 3:** List of the 9 most significant small molecule drugs that can reverse the tumoral status of LSCC

CMap name	Mean	N	Enrichment	*P*	Specificity	Percent non-null
Thiocolchicoside	− 0.85	4	− 0.837	0.00123	0	100
Gibberellic acid	− 0.714	4	− 0.804	0.00284	0.0107	100
Ciclacillin	− 0.866	4	− 0.786	0.00422	0.0056	100
Cetirizine	− 0.705	4	− 0.781	0.00465	0.0242	100
Medrysone	− 0.7	6	− 0.764	0.00032	0.0108	100
Lomefloxacin	− 0.655	6	− 0.726	0.00085	0	83
Pramocaine	− 0.708	5	− 0.711	0.00435	0	80
Metamizole sodium	− 0.695	6	− 0.657	0.00455	0.0684	83
Tolbutamide	− 0.566	7	− 0.598	0.00643	0	71

### Chromogenic in situ hybridization (CISH)

**Fig. 10:**
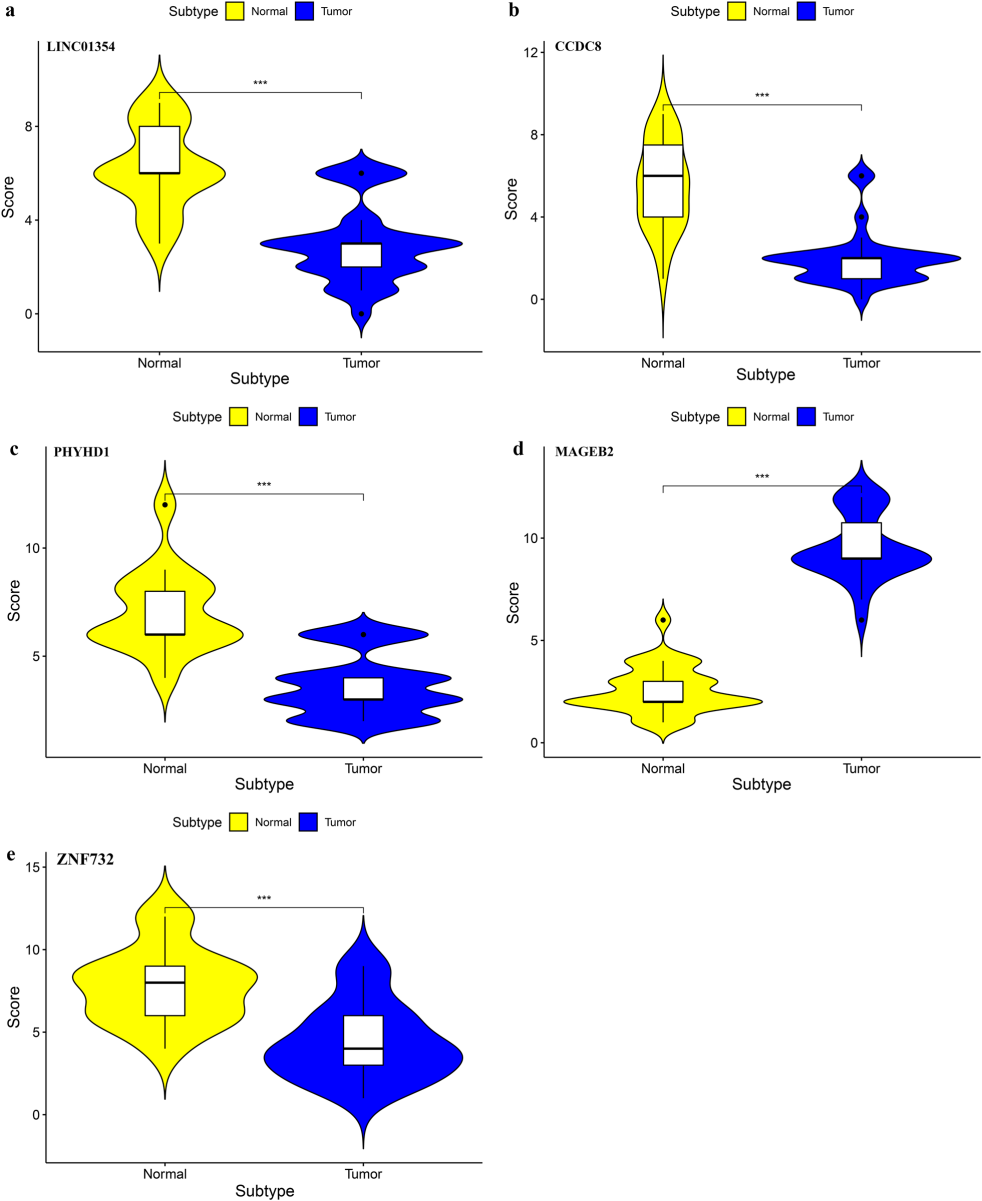
CISH staining score analysis showed that LINC01354, CCDC8, PHYHD1, and ZNF732 are downregulated in LSCC compared to expression in adjacent normal mucosa tissues (**a**–**c**, **e**); MAGEB2 is upregulated in LSCC compared to expression in adjacent normal mucosa tissues (**d**); ***P < 0.001

The expression of LINC01354, CCDC8, PHYHD1, MAGEB2 and ZNF732 in 30 paraffin-embedded tissue samples of LSCC and adjacent non-neoplastic tissues was semiquantitatively examined by CISH (Fig. 9). Staining score analysis revealed that MAGEB2 has significantly higher expression of LSCC compared to adjacent non-neoplastic tissues (Fig. 10d); LINC01354, CCDC8, PHYHD1, and ZNF732 have significantly lower expression of LSCC compared to adjacent non-neoplastic tissues (Fig. 10a, c, e), which were in line with bioinformatics analysis results.

## Discussion

Integrating TCGA LSCC RNA-seq datasets with DNA methylation datasets by MethylMix tools, we identified 88 DNA MDGs. On the basis of these DNA MDGs, we developed a RFS-related MDGPS, which could accurately categorized patients into high-risk status and low-risk status. Stratification analysis verified that the MDGPS remained a significant statistical prognostic model in subsets of patients with different clinical variables. Multivariate Cox regression analysis uncovered the efficacy of MDGPS appears independent of other clinicopathological characteristics. With respect to predictive capacity and clinical usefulness, the MDGPS was superior to traditional TNM stage. Additionally, the MDGPS was validated in external LSCC cohorts from GEO. Finally, CMap matched the 9 most significant small molecules as promising therapeutic drugs to reverse the LSCC gene expression.

Increasing number of researches recognizes that epigenetic changes such as the hypermethylation of tumor suppressor genes and hypomethylation of oncogenes in the diagnosis, progression and prognosis of LSCC played a critical role [11–13]. By quantitative methylation-specific polymerase chain reaction (qMSP) assays in 96 LSCC patients, Shen et al. [11] revealed that LZTS2 promoter hypermethylation is linked to risk, progression, and prognosis of LSCC, which can serve as diagnostic and prognostic biomarker for LSCC. Wang et al. [12] uncovered that in laryngeal cancer cells demethylated SP1 sites in CG-rich region of miR-23a-27a-24–2 cluster promoter upregulate the cluster expression, resulting in early apoptosis inhibition and proliferation promotion probably via targeting the related targets such as PLK2 and APAF-1.On the basis of 77 LSCC patients, Liu et al. [13] found that hypermethylation percentage RUNX3 was associated with lymph node metastasis,TNM classification of malignant tumors stage, poor OS rate as well as suppression of RUNX3 expression. Similarly, in vitro reduced methylation and increased expression of RUNX3 genes was confirmed and following 5-azacytidine treatment, decreased cell migration was observed. RUNX3 promoter region may be a potential therapeutic spot for LSCC.

These studies indicated the potential clinical implications of DNA methylation in offering new biomarker for valuable molecular targeted therapy and establish predictive models to optimize therapeutic strategies in LSCC patients. However, most of studies define overall survival (OS) as primary outcome, not for RFS. Because OS is more likely to be affected by comorbidity and post-recurrence treatment, RFS could more truly reflect the biologic behavior for LSCC patients [14–16]. Besides, almost all of researches focused on the methylation status of one gene with limited statistical power in predictive values. Considering the heterogeneity of LSCC, entire molecular signatures derived from high-content genome screens seem to offer better prognostic value.

To our knowledge, this is the first study carried out a genome-wide integrated analysis of methylation and the transcriptome from TCGA database to create an MDGPS for LSCC patients to optimize therapeutic strategies and seek novel biomarker as potential molecular targeted therapy. When applying high-throughput methodology with 450,000 probes, it is necessary to distinguish the epigenetic changes (“driver”) that act as effectors of the malignant phenotype from alterations of “passenger” without any biologic function [17]. Hence, a model-based tool (MethylMix) is an attractive investigative tool to integrate DNA methylation with RNA expression to identify MDGs in LSCC, which focuses on identifying cis-regulatory effects of DNA methylation on gene expression. Consequently, we identified a cohort of 88 MDGs in LSCC. The functional analysis indicated MDGs were mainly attached oneself to gene expression (transcription), RNA polymerase II transcription, transcription factor activity, sequence-specific DNA binding and so on. It was a hint that DNA methylation is involved in the dysregulation of genes with distinct functions and is functionally linked to outcomes in LSCC patients.

On the basis of univariate and multivariate Cox regression analyses, we selected five MDGs (LINC01354, ZNF732, CCDC8, PHYHD1 and MAGEB2) to develop a RFS-related MDGPS. It could effectively classified patients into high-risk group with shorter RFS and low-risk group with longer RFS in TCGA and GEO database. Remarkably, according to stratified analysis, the MDGPS was a statistically and clinically prognostic model in lymphovascular invasion or no lymphovascular invasion, negative margin status, G1–G2 or G3–G4, III–IV stage, N0 or N1-N3, T3-T4 subgroup. But, in positive margin status, I-II stage and T1–T2 subgroup did not reach significant statistic. Yet, in positive margin status, I-II stage and T1–T2 subgroup did not reach significant statistic. One possible explanation that a small sample size of T1–T2 or I–II stage or positive margin status subgroup, consisting of less than 10 patients in different subgroup, are not enough to generate an effect of significant statistics. In addition, univariate and multivariate Cox analysis affirmed that MDGPS was an independent predictor of unfavorable RFS, regardless of other clinicopathologic variables in TCGA and GEO data set. To explore potential biological pathways for 5 RFS-related MDGs, we carried out the GSEA and indicated that 5 MDGs of MDGPS was mainly scattered in cancer-related pathways (ErbB signaling pathway, mTOR signaling pathway, MAPK signaling pathway, Notch signaling pathway), metabolism-related pathways(calcium signaling pathway, RNA degradation) and immune-related biological processes (T cell receptor signaling pathway). It implied that 5 MDGs of MDGPS maybe involved in initiation, maintenance, development of LSCC and associated with outcomes in LSCC patients.

At present, the AJCC (American Joint Committee on Cancer) TNM (tumor-node -metastasis) stage system [18], on the basis of anatomical information, is often utilized when talking about decision making about treatment for LSCC patients. Nevertheless, LSCC is composed of heterogeneous histologic subtypes with a wide range of clinical course variations. Consequently, a significant proportion of patients with inaccurate stage maybe receive over-treatment or inadequate treatment. For example, over-stage might subject a patient to needless adjunctive chemoradiotherapy; conversely, under-stage is likely to result in recurrence or even death after surgery. In our research, via ROC curve analysis, MDGPS shown more precise predictive ability compared with TNM stage model in TCGA and GEO database, which could effectively identify high-risk patients prone to adjunctive chemoradiotherapy and low-risk to avoid needless adjuvant therapy. Interestingly, DCA results indicated that LSCC recurrence-related treatment decision based on the MDGPS resulted in more net benefit than treatment decision based on TNM stage, or treating either all patients or none in TCGA and GEO database. To sum up, the current MDGPS would be clinically useful for the clinicians in tailoring recurrence-associated treatment decision.

One prominent finding in our study was that combining methylation and RNA expression data with survival analysis, we identified 4 MDGs (LINC01354, CCDC8, PHYHD1 and MAGEB2), which maybe serve as potential biomarkers or drug targets for early diagnosis and prognostic assessment. LINC01354, a long non-coding RNAs (lncRNAs), play critical roles in tumor progression. Li et al. [19] revealed that high level of LINC01354 expression is closely related to distant metastasis, lymph node metastasis, tumor size, and TNM stage in colorectal cancer. Functional analysis uncovered that LINC01354 promoted colorectal cancer cell migration, proliferation, and epithelial-mesenchymal transition. Mechanistically, LINC01354 stabilized CTNNB1 via interacting with hnRNP-D, thereby leading to activation of Wnt/β-catenin signaling pathway. It was recently reported that dysregulation of LINC01354 in thyroid cancer is associated with genomic alterations [20]. CCDC8 (coiled-coil domain containing 8) encodes a helical domain containing protein, which is one of three proteins mutated in 3 M syndrome patients [21]. Findings by Li et al. [22] confirmed that epigenetic dysregulation of CCDC8 may result in metastasis to the brain as well as other distant organs in breast tumors. During tumor evolution, CCDC8 dysregulation early occurs, which has the potential to be utilized as a prognostic marker in addition to being a potential therapeutic target. PHYHD1 (phytanoyl-CoA dioxygenase domain containing 1), which is a putative orthologue of Xenopus phytanoyl-CoA dioxygenase-like (XPhyHlike) [23]. Nevertheless, information regarding the role of PHYHD1 in cancer is lacking. MAGEB2, as a member of the MAGEB family, which belongs to the cancer testicular antigens, is located in the last exon on chromosome X. The MAGEB2 gene has been reported to be overexpression in several types of tumors, such as lung cancer [24] and malignant peripheral nerve sheath tumors [25], which has been implicated in carcinogenesis and considered as a potential cancer biomarker [26]. Pattini et al. [27] indicated that MAGEB2 is activated by promoter demethylation in head and neck squamous cell carcinoma (HNSCC), which has growth promoting effects on a minimally transformed oral keratinocyte cell line. Thus, further characterization of molecules such as LINC01354, CCDC8, PHYHD1 and MAGEB2 will provide new perspective for the development and progress of LSCC, and aided to find potential therapeutic targets for LSCC patients.

Another finding in our study was that we identify a set of potential small molecule drugs that reverse abnormal gene expression of LSCC, analyzing the MDGs in CMap database. Small molecule drugs with a highly significant negative enrichment value possessed the potential to alter the gene expression of LSCC, and thus inhibiting the progression of tumors. Thiocolchicoside, a semi-synthetic colchicine derived from plant honeysuckle, is a muscle relaxant and utilized to treat orthopedic disorders and rheumatologic on account of its anti-inflammatory and analgesic mechanisms. Reuter et al. [28] demonstrate that thiocolchicoside exert an effect on anticancer via the NF-κB pathway resulting in inhibition of cyclooxygenase-2 promoter activity and NF-κB reporter activity. However, efficacy and safety of those small molecule drugs on LSCC are still not investigated. Hence, it is urgently demanded to verify the effect of these candidate small molecule drugs on treating LSCC in the further experiments.

Despite the remarkable sense, it is inevitable that limitations also existed in our study. First, we merely extract retrospectively target data (TCGA and GEO datasets) through biological algorithm approaches absence of fresh clinical samples to screen and verify our results. So, the application of our model remains needed to validate in external and multicenter prospective cohorts with large sample sizes. Second, the 5 MDGs should be further studied and verified to investigate its specific regulatory function and mechanisms in LSCC. Third, the molecular profiling of the tumors as presented here is that it might be inclined to intra-tumor heterogeneity, not display spatial pattern of biomarker expression (including focal sub-clones) across the tumor specimen. Therefore, the future direction of methylation and expression profiling in tumor risk stratification will require a single cell based approach.

## Conclusion

A MDGPS, with five DNA MDGs, was identified and validated in LSCC patients by integrating multidimensional genomic data. Compared TNM stage alone, it generates more accurate estimations of the survival probability and maybe help the development of personalized and precise medicine LSCC field.

## Supplementary information

**Additional file 1: Material S1.** Clinical information data of 117 LSCC samples from TCGA database.

**Additional file 2: Material S2.** Clinical information data 109 LSCC specimens from GSE27020 database.

**Additional file 3: Material S3.** Clinical information data 109 LSCC specimens from GSE25727 microarray.

**Additional file 4: Table S1.** Detailed information of different R packages for analysis.

**Additional file 5: Material S4.** Identification of 88 methylation-driven genes with Cor <−0.3, |logFC| > 0and P < 0.05.

**Additional file 6: Figure S1.** Gene ontology analysis of 88 MDGs of LSCC.

**Additional file 7: Figure S2.** Functional pathway analysis for 88 MDGs based on ConsensusPathDB database. Only the pathways which ***P*** < 0.05 were shown here. Node size: the number of genes; Node color: P-value; Edge width: percentage of shared genes; Edge color: genes from input.

**Additional file 8: Figure S3.** Distribution map of the methylation degree of the five MDGs in the MDGPS. The X‐axis indicates the degree of methylation, the Y‐axis indicates the number of methylated samples, and the histogram shows the subgroups of the methylation distribution in the cancer group. The black horizontal line indicates the methylation degree distribution of the normal samples. The simulated trend of methylation distribution in cancer tissues is represented by the curve.

**Additional file 9: Figure S4.** Correlation between the methylation degree and expression of the five MDGs in the MDGPS. The X‐axis indicates the gene methylation degree β value, and the Y‐axis indicates the gene expression level.

**Additional file 10: Figure S5.** Kaplan–Meier survival analysis according to the MDGPS stratified by clinicopathological factors. (A) T stage- T1 to T2, (B) T stage- T3 to T4; (C) lymph node status-node negative, (D) lymph node status-node positive; (E) TNM stage- stage I-II, (F) TNM stage- stage III-IV.

**Additional file 11: Figure S6.** Kaplan–Meier survival analysis according to the MDGPS stratified by clinicopathological factors. (A) Tumor grade—G1 to G2, (B) Tumor grade—G3 to G4; (C) margin status- negative, (D) margin status—positive; (E) lymphovascular invasion-no, (F) lymphovascular invasion-yes.

**Additional file 12: Figure S7.** Kaplan–Meier survival curves for the joint survival analysis-the combination of gene ZNF732 methylation and expression.

**Additional file 13: Table S2.** Screening of 16 prognostic risk loci associated with MDGs in LSCC.

**Additional file 14: Material S5.** Matching 3587 small molecules drugs by CMap database.

## Data Availability

The data that support the findings of this study are provided in additional materials and are also made available in the TCGA (https://gdc.cancer.gov/) and GEO (https://www.ncbi.nlm.nih.gov/geo/).

## Abbreviations

LSCC: Laryngeal squamous cell carcinoma
MDGPS: Methylation-driven genes prognostic signature
RNA-seq: RNA sequencing
TCGA: The Cancer Genome Atlas
MDGs: Methylation-driven genes
ROC: Receiver operating characteristic
AUC: Area under curve
DCA: Decision curve analysis
GEO: Gene Expression Omnibus
CISH: Hromogenic in situ hybridization
RFS: Recurrence-free survival
OS: Overall survival
GO: Gene ontology
MF: Molecular function
BP: Biological process
CC: Cellular component
LNR: Lymph node ratio
LNs: Lymph nodes
qMSP: Quantitative methylation-specific polymerase
AJCC: American Joint Committee on Cancer
TNM: Tumor-node-metastasis
CCDC8: Coiled-coil domain containing 8
PHYHD1: Phytanoyl-CoA dioxygenase domain containing 1
HNSCC: Head and neck squamous cell carcinoma
DIG: Digoxigenin
