# Supplementary material for: Identification and validation of methylation-driven genes prognostic signature for recurrence of laryngeal squamous cell carcinoma by integrated bioinformatics analysis
Source: Cancer Cell Int. 2020 Sep 29;20:472. doi: 10.1186/s12935-020-01567-3 (PMC7526132; doi:10.1186/s12935-020-01567-3)
Supplement: Supplementary file 4 — Additional file 4: Table S1. Detailed information of different R packages for analysis. [file 12935_2020_1567_MOESM4_ESM.docx]

**TableS1.** [Detailed](javascript:;) [information](javascript:;) of different R packages for analysis.

| Objective | Method | Package name |
| --- | --- | --- |
| Normalize the transcriptome data | NA | edgeR package |
| Normalize the methylation data | NA | Limma package |
| Identifcation of DNA methylation‑driven genes | Original manuscript (three steps) | MethylMix R package |
| Select RFS-related MDGs | univariable Cox regression analysis | Survival R package |
| Uncovering biological meaning of genes | Gene ontology (GO) analysis | GOplot R package |
| Select RFS-related MDGs | multivariate Cox regression model | Survival R package |
| Differences in survival between the two groups | Kaplan-Meier survival analysis | Survival R package |
| Predictive performance  in different times | Time-dependent receiver operating characteristic | TimeROC package |
| Comparative studies  in different models or factors | ROC analysis | SurvivalROC package |
| Clinical usefulness | Decision curve analysis | stdca.R |
| NA | Stratified analysis | Survival R package |
| NA | Joint survival analysis | Survival R package |

Abbreviations: RFS= recurrence-free survival; MDGs= methylation‑driven genes; NA= not available; ROC= receiver operating characteristic
