# Supplementary figures and images for: Identification and validation of methylation-driven genes prognostic signature for recurrence of laryngeal squamous cell carcinoma by integrated bioinformatics analysis
Source: Cancer Cell Int. 2020 Sep 29;20:472. doi: 10.1186/s12935-020-01567-3 (PMC7526132; doi:10.1186/s12935-020-01567-3)

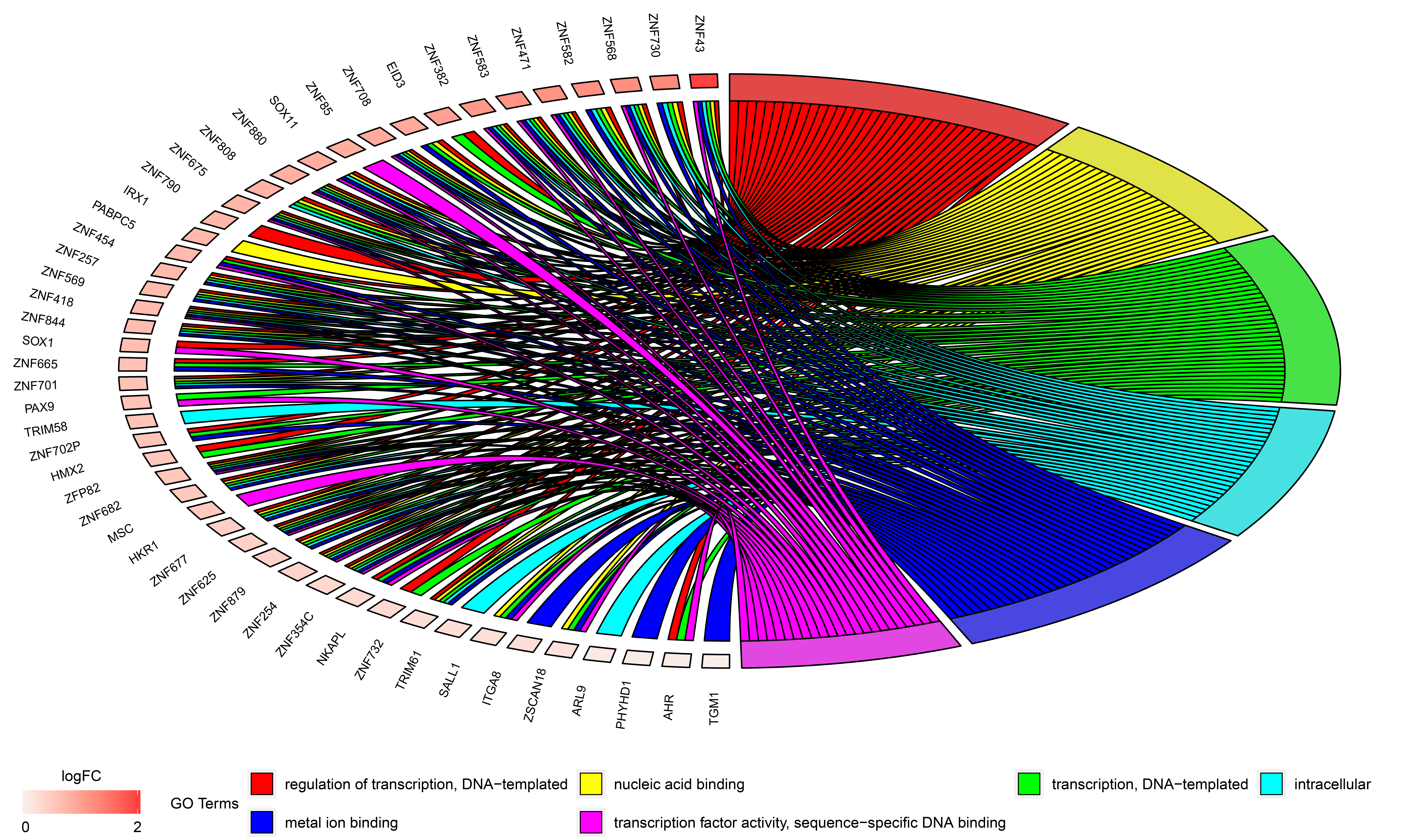

Supplement: Supplementary file 6 — Additional file 6: Figure S1. Gene ontology analysis of 88 MDGs of LSCC. [file 12935_2020_1567_MOESM6_ESM.tif]

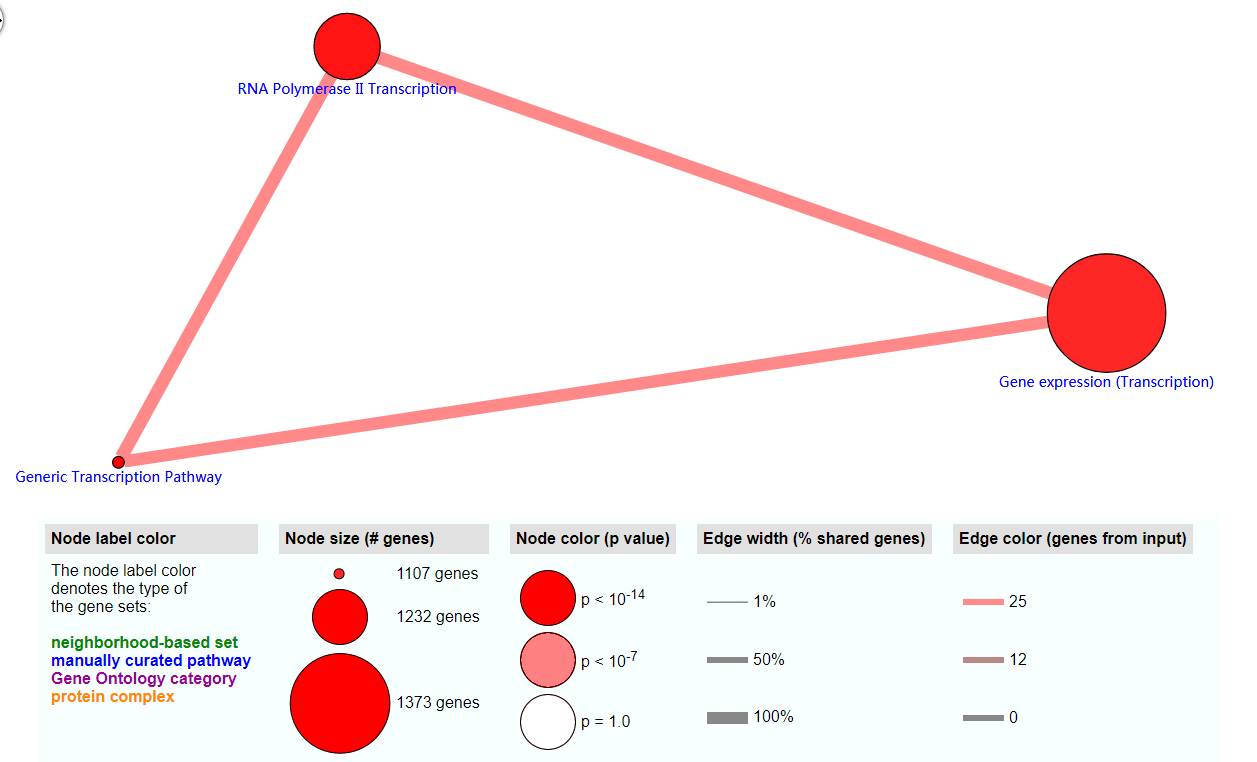

Supplement: Supplementary file 7 — Additional file 7: Figure S2. Functional pathway analysis for 88 MDGs based on ConsensusPathDB database. Only the pathways which P < 0.05 were shown here. Node size: the number of genes; Node color: P-value; Edge width: percentage of shared genes; Edge color: genes from input. [file 12935_2020_1567_MOESM7_ESM.tif]

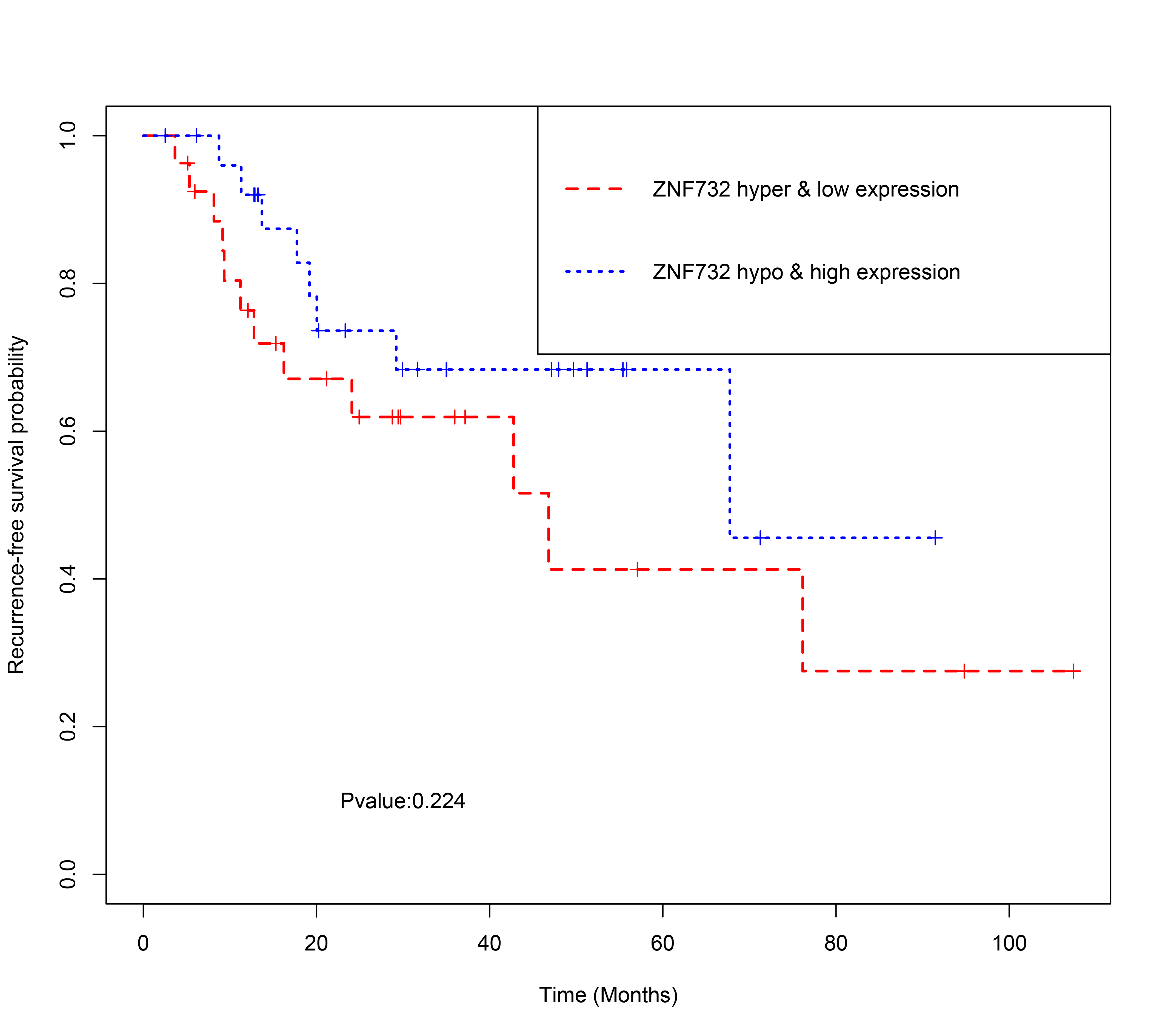

Supplement: Supplementary file 12 — Additional file 12: Figure S7. Kaplan–Meier survival curves for the joint survival analysis-the combination of gene ZNF732 methylation and expression. [file 12935_2020_1567_MOESM12_ESM.tif]
