# Supplementary material for: Identification and validation of methylation-driven genes prognostic signature for recurrence of laryngeal squamous cell carcinoma by integrated bioinformatics analysis
Source: Cancer Cell Int. 2020 Sep 29;20:472. doi: 10.1186/s12935-020-01567-3 (PMC7526132; doi:10.1186/s12935-020-01567-3)
Supplement: Supplementary file 13 — Additional file 13: Table S2. Screening of 16 prognostic risk loci associated with MDGs in LSCC. [file 12935_2020_1567_MOESM13_ESM.docx]

**TableS2.** Screening of 16 prognostic risk loci associated with MDGs in LSCC.

| Risk methtylated locus | Related genes | Hazard ratio | *P* **value** |
| --- | --- | --- | --- |
| cg10811426 | **LINC01354** | 76.91421876 | 0.01399869 |
| cg21546522 | **LINC01354** | 37.17321982 | 0.02220425 |
| cg05133706 | **LINC01354** | 19.88259653 | 0.02901080 |
| cg00123090 | **LINC01354** | 14.99485287 | 0.03777544 |
| cg12657297 | **LINC01354** | 10.23917435 | 0.04958551 |
| cg15984661 | **CCDC8** | 11.77786536 | 0.01097912 |
| cg18653451 | **CCDC8** | 10.23602828 | 0.02029739 |
| cg03576469 | **CCDC8** | 6.543773047 | 0.02042613 |
| cg06747432 | **CCDC8** | 6.82639136 | 0.02140057 |
| cg11715966 | **CCDC8** | 23.21802173 | 0.02498201 |
| cg12754854 | **PHYHD1** | 406.0949758 | 0.00660412 |
| cg13022129 | **PHYHD1** | 81.68997846 | 0.01477033 |
| cg13367612 | **PHYHD1** | 207.779827 | 0.02061176 |
| cg14153069 | **PHYHD1** | 28.05874196 | 0.02481943 |
| cg13613439 | **PHYHD1** | 22.80881197 | 0.03325798 |
| cg17299712 | **MAGEB2** | 0.102412578 | 0.04941135 |
